# Supplementary material for: A tactile discrimination task to study neuronal dynamics in freely-moving mice
Source: Nat Commun. 2025 Jul 11;16:6421. doi: 10.1038/s41467-025-61792-0 (PMC12254278; doi:10.1038/s41467-025-61792-0)
Supplement: Supplementary file 1 — Supplementary Information [file 41467_2025_61792_MOESM1_ESM.pdf]

Supplementary Materials

A tactile discrimination task to study neuronal dynamics in freely-moving mice

Filippo Heimburg*[,1], Nadin Mari Saluti*[,1], Josephine Timm*[,1,2], Avi Adlakha [3], Maria Helena Bortolozzo-Gleich[1], Jesús Martín-Cortecero[1], Melina Castelanelli[1], Matthias Klumpp[1], Lee Embray[1], Martin Both[1], Thomas Kuner[3] and Alexander Groh[1]

*These authors contributed equally
[1] Institute for Physiology and Pathophysiology, Heidelberg University, 69120, Germany
[2] Current address: Institute for Experimental Epileptology and Cognition Research, University of Bonn, 53105, Germany
[3] Institute for Anatomy and Cell Biology, Heidelberg University, 69120, Germany
groh@uni-heidelberg.de (AG)

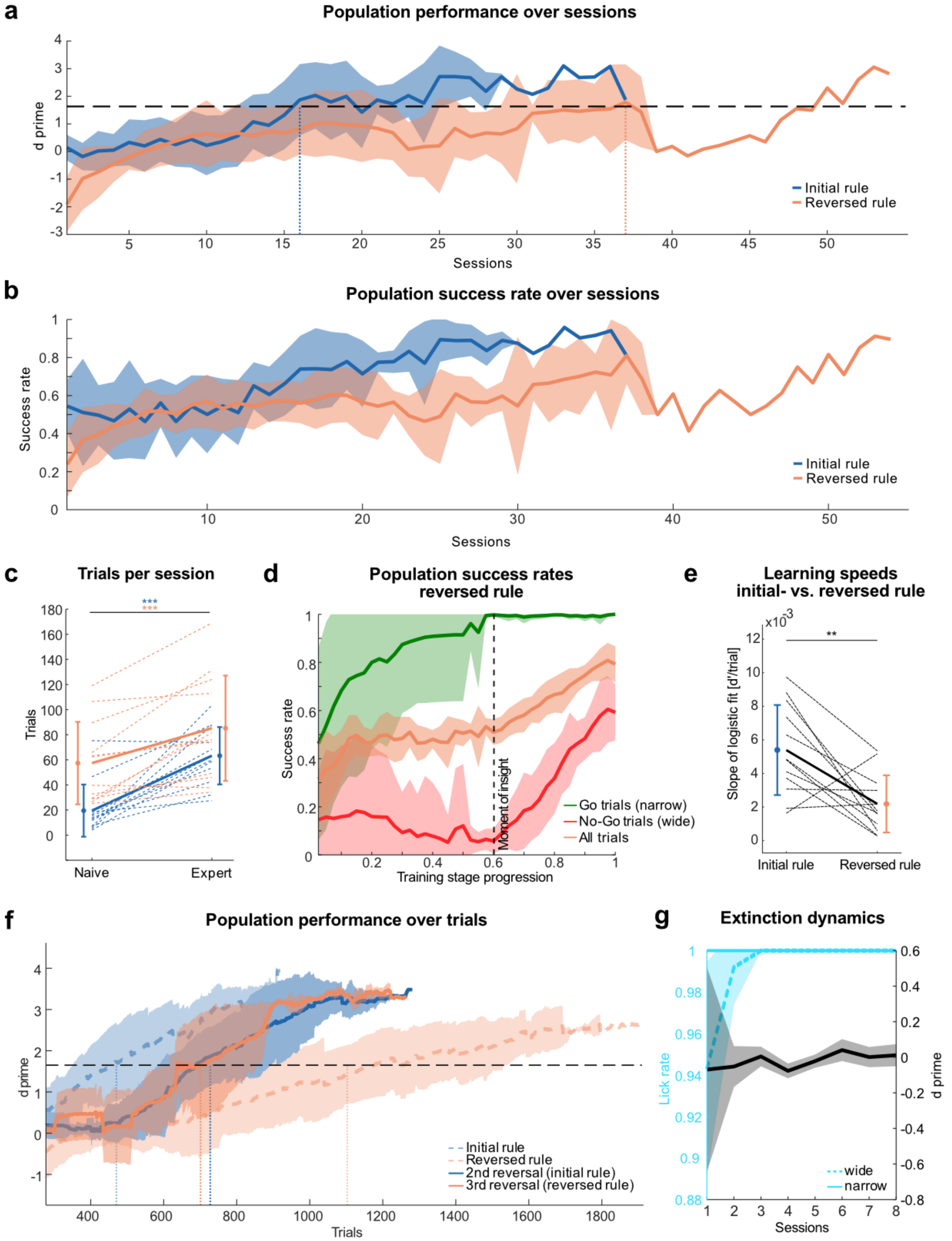
a
Population performance over sessions
d prime
Sessions
Initial rule
Reversed rule
b
Population success rate over sessions
Success rate
Sessions
Initial rule
Reversed rule
c
Trials per session
Trials
Naive
Expert
d
Population success rates reversed rule
Success rate
Training stage progression
Moment of insight
Go trials (narrow)
No-Go trials (wide)
All trials
e
Learning speeds initial- vs. reversed rule
Slope of logistic fit [d'/trial]
Initial rule
Reversed rule
f
Population performance over trials
d prime
Trials
Initial rule
Reversed rule
2nd reversal (initial rule)
3rd reversal (reversed rule)
g
Extinction dynamics
Lick rate
d prime
Sessions
wide
narrow

<u>Supplementary Fig. 1: Learning curves for initial and repeated rule reversals.</u> **a:** Population performance over sessions for a contrast of 20 mm. Solid lines show mean d'; shaded areas show standard deviation (horizontal dashed line: expert-level performance threshold (d' = 1.65); n = 12 mice). Animals reached expert-level performance after 16 sessions in the initial rule stage and after 37 sessions in the reversed rule stage (dotted vertical lines). **b:** Population success rates over sessions for a contrast of 20 mm. Solid lines show mean d'; shaded areas show standard deviation (n = 12 mice). **c:** Trials per session during the initial and reversed rule stage for naive and expert animals (dotted lines: paired values of individual animals; solid line: paired values of population means; data points with error bars: population means and standard deviations; n = 12 mice). **d:** Success rates over stage progression for Go- (Hit/(Hit+Miss)), No-go (CR/(CR+FA)) and all trials ((Hit+CR)/(Hit+Miss+CR+FA)) (n = 12 mice). Dashed line indicates "moment of insight", defined by a jump in success rate in no-go trials by a factor of five from one session to the next (n = 7 mice).**e:** Learning speeds for initial and reversed rules (same conventions as in b; n = 12 mice). **f:** Population performance over trials for initial-, 1st- 2nd- and 3rd-rule reversal. Mean d' calculated with a running window of the preceding 200 trials; shaded areas show standard deviation (horizontal dashed line: expert-level performance threshold (d' = 1.65); n = 12 mice for initial and reversed, 5 mice for 2nd reversal, 2 mice for 3rd reversal). Animals reached expert-level performance after 388 trials in the initial rule stage, after 1077 trials in the reversed rule stage, after 728 trials in the 2nd reversal and after 701 trials in the 3rd reversal (dotted vertical lines). **g:** Mean lick rates (wide (previous No-go), narrow (previous Go)) and d prime over extinction sessions (n = 5 mice).

ns: $p > 0.05$, *: $p \leq 0.05$, **: $p \leq 0.01$ and ***: $p \leq 0.001$; **a:** two-tailed Wilcoxon rank sum test, **d, e:** paired t-test. For exact statistics see Supplementary Table 1.

.

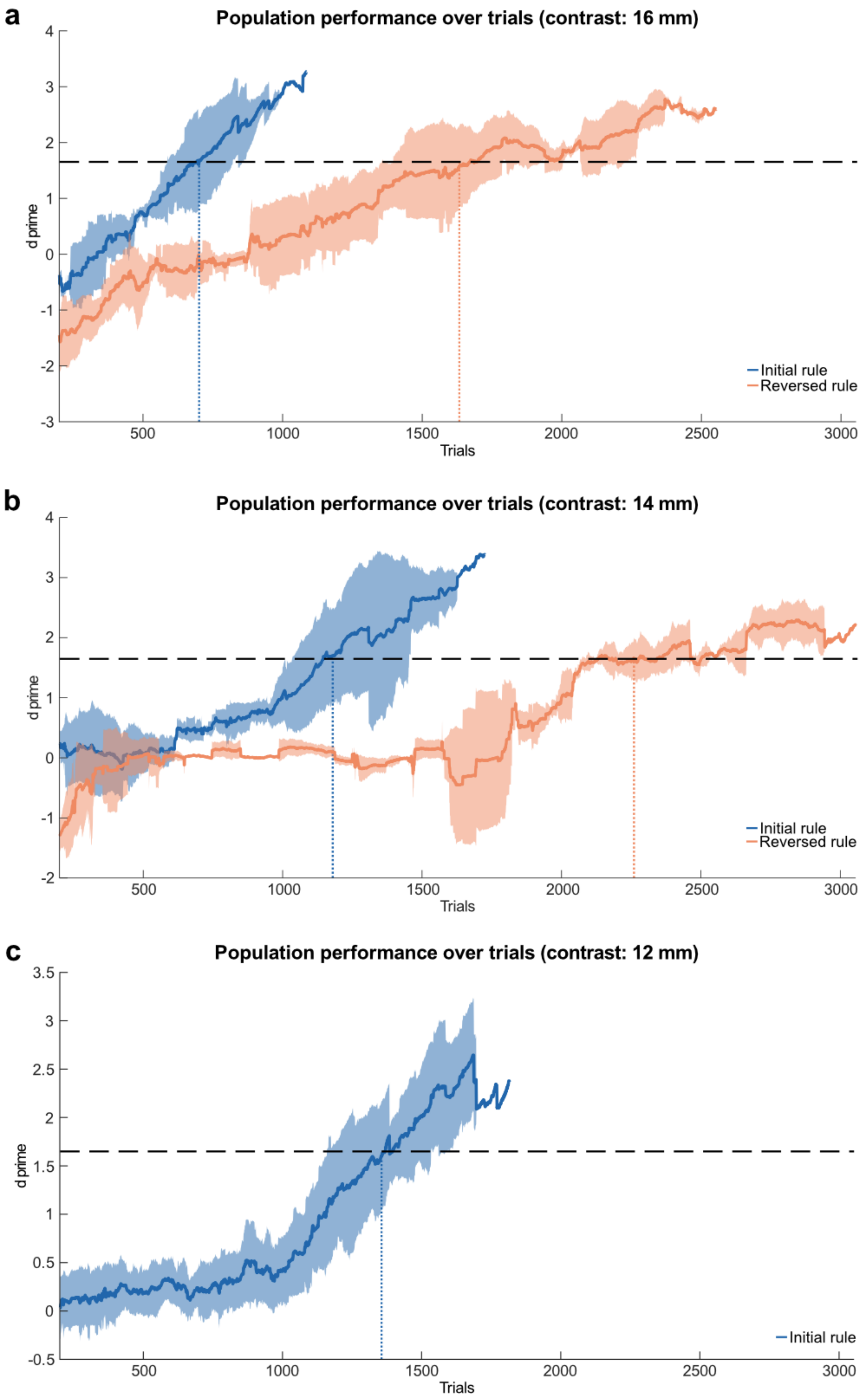
a
Population performance over trials (contrast: 16 mm)
d prime
Trials
Initial rule
Reversed rule
b
Population performance over trials (contrast: 14 mm)
d prime
Trials
Initial rule
Reversed rule
c
Population performance over trials (contrast: 12 mm)
d prime
Trials
Initial rule

Supplementary Fig. 2: Learning curves for different contrasts. **a:** Population performance over trials for 16 mm contrast. Solid lines show mean d', calculated with a running window of the preceding 200 trials; shaded areas show standard deviation (initial rule: blue and reversed rule: orange; horizontal line: expert-level performance threshold (d' = 1.65); n = 3 mice). Animals reached expert-level performance after 701 trials in the initial rule stage and after 1633 trials in the reversed rule stage (dotted vertical lines). **b:** Same as in A for 14 mm contrast (n = 3 mice (initial rule) and 2 mice (reversed rule)). Animals reached expert-level performance after 1180 trials in the initial rule stage and after 2260 trials in the reversed rule stage (dotted vertical lines). **c:** Same as in A, B for 12 mm contrast (n = 6 mice). Animals reached expert-level performance after 1356 trials in the initial rule stage (dotted vertical line).

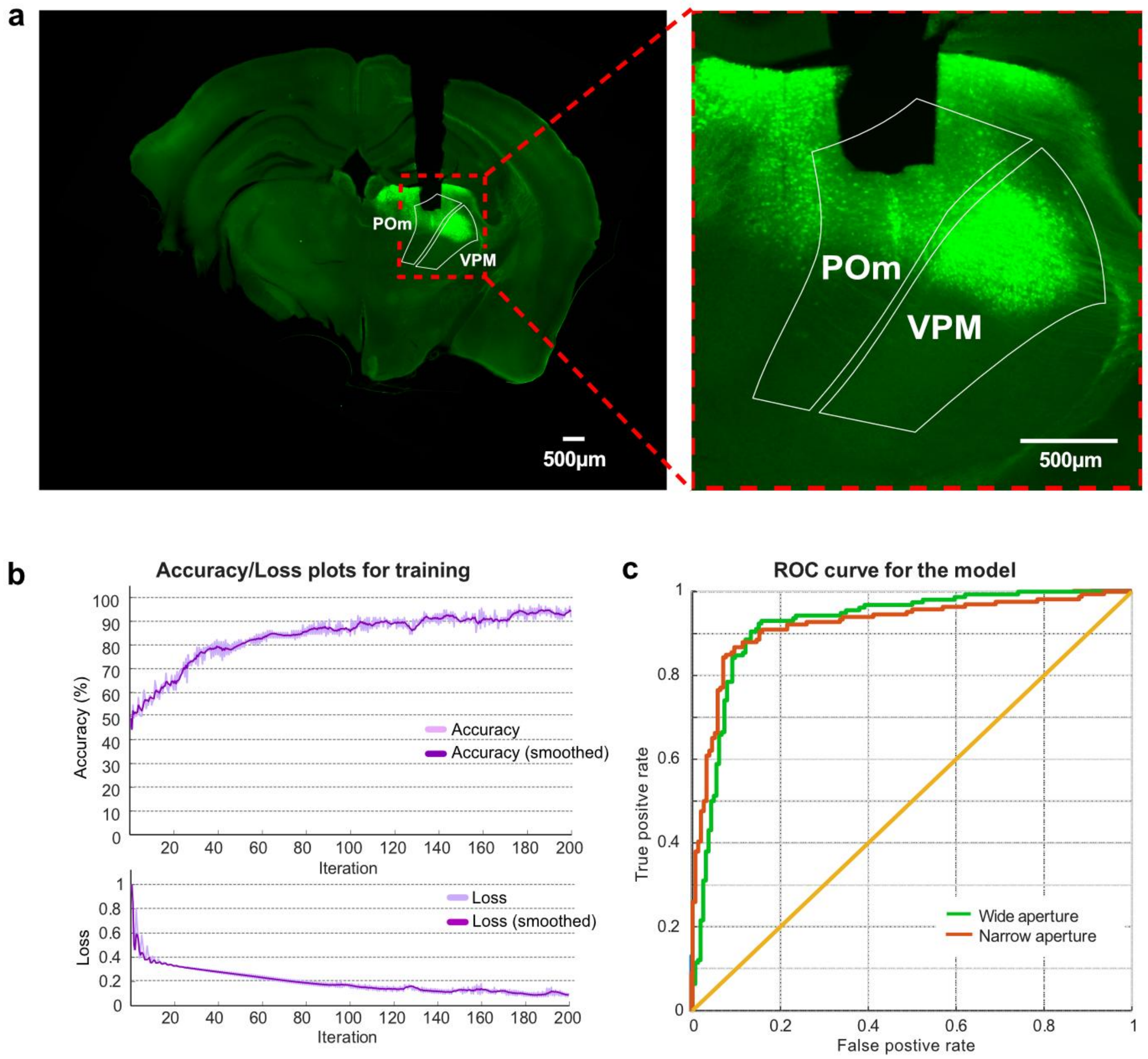

Supplementary Fig. 3: Miniscope imaging during aperture discrimination learning task. **a:** Left: Coronal section of example mouse brain slice, indicating the tract of the GRIN lens, and the recording region for GCamp6f (green) calcium imaging in POm. Right: same as left at higher magnification. **b:** The convolutional neural network (CNN) classifier for aperture width using calcium imaging data. Shown are Accuracy/Loss plots for the training of the CNN classifier. **c:** The ROC statistic curve for classification of the two apertures.

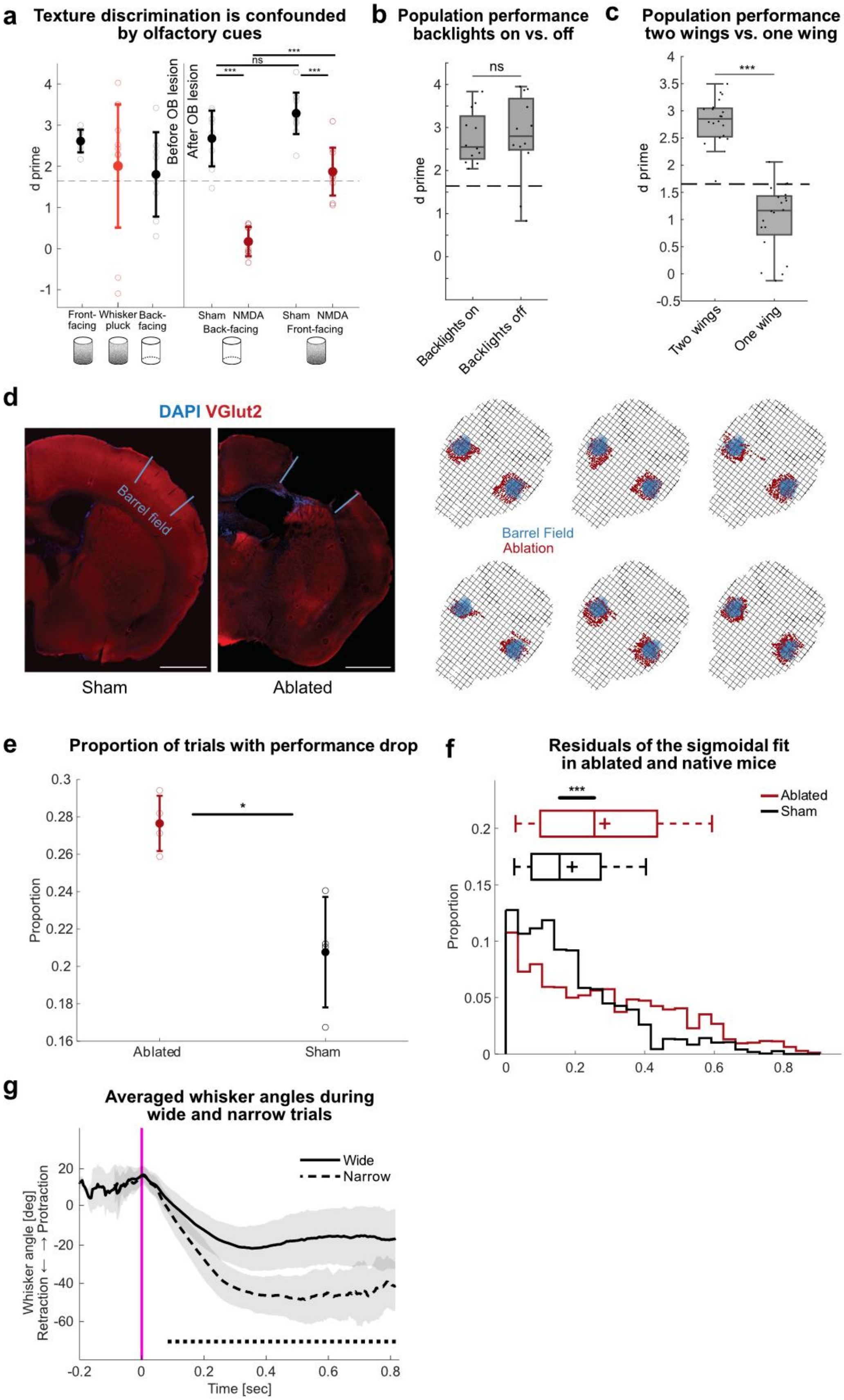

a
Texture discrimination is confounded by olfactory cues
Before OB lesion
After OB lesion
d prime
Front-facing
Whisker pluck
Back-facing
Sham
NMDA
Back-facing
Sham
NMDA
Front-facing
***
ns
***
***
b
Population performance backlights on vs. off
ns
d prime
Backlights on
Backlights off
c
Population performance two wings vs. one wing
***
d prime
Two wings
One wing
d
DAPI VGlut2
Barrel field
Sham
Ablated
Barrel Field
Ablation
e
Proportion of trials with performance drop
*
Proportion
Ablated
Sham
f
Residuals of the sigmoidal fit in ablated and native mice
***
Ablated
Sham
Proportion
g
Averaged whisker angles during wide and narrow trials
Wide
Narrow
Whisker angle [deg]
Retraction ← → Protraction
Time [sec]

Supplementary Fig. 4: Increased performance variability in ablated mice. **a:** Performance before and after olfactory bulb (OB) lesion with NMDA (red) or sham injections with saline (black) during sandpaper texture discrimination (n = 2 mice). Before OB lesion, mice discriminated both the-front facing and inverted (back-facing) textures with similar accuracy. Following OB lesions, performance was completely abolished, while sham-lesioned mice retained discrimination ability. Performance was restored when sandpapers were presented in their original orientation (front-facing. Each dot represents an individual session, with mean ± standard deviation shown. **b:** Performance of expert animals with backlights on and off. Horizontal dashed line indicates expert-level performance threshold (d' = 1.65). Performance was calculated from the last two sessions with backlights on and the first two sessions with backlights off, each dot represents one session. **c:** Performance of expert mice (n = 5 mice) with two aperture wings or one aperture wing. Performance was calculated from a total of 20 sessions across all mice for each condition. **d:** Barrel cortex ablations: Coronal sections (60µm) of eight ablated brains were imaged, and the barrel field was visualized using an anti-vGlut2 stain (left panel: example of a sham and ablated section). The extent of the barrel field (blue) and the ablation (red) were annotated in each section. Sections and annotations were then registered and visualized using the Allen CCF framework (https://github.com/cortex-lab/allenCCF) for all eight ablated brains (right panel). **e:** Proportion of trials that were followed by a performance drop in ablated (red) and control (black) mice. Ablated mice exhibited a significantly greater proportion of trials associated with performance drops compared to controls. Each dot represents an individual mouse, with error bars indicating mean ± 95% CI (0.28±0.01 for ablated mice; 0.21±0.03 for control mice). **f:** Distribution of residuals from the sigmoidal fit to trial performance in ablated (red) and control (black) mice. Ablated mice exhibited significantly larger residuals, indicative of heightened performance variability. Box plots display the median (central line) and mean (cross). **g:** Average whisker angles upon encountering the wide (solid) or narrow (dashed) aperture, with mean ± standard deviation. Horizontal dashed lines indicate significant differences.

ns: $p > 0.05$, *: $p \leq 0.05$, **: $p \leq 0.01$ and ***: $p \leq 0.001$; **a:** two-tailed Wilcoxon rank sum test, **b:** paired t-test, **c:** repeated measures anova, **d, f:** two-tailed Wilcoxon rank sum test, **g:** Welch's t-test; if not stated otherwise, data was pooled from n = 6 mice. For exact statistics see Supplementary Table 1.

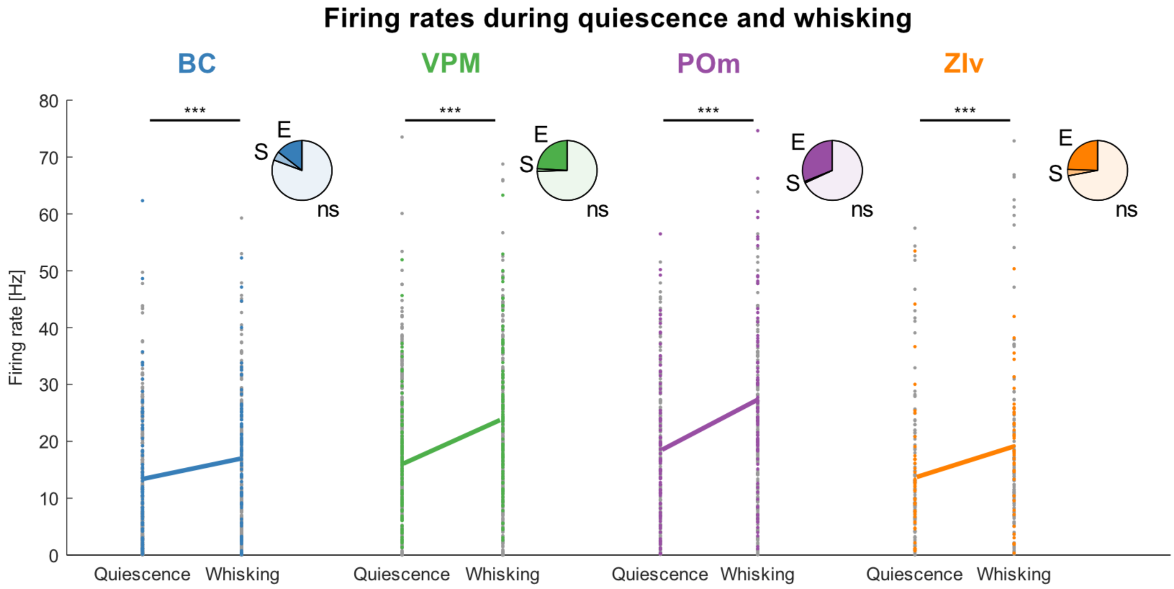

Supplementary Fig. 5: Firing rates during quiescence and whisking. Firing rates during quiescence and whisking across four brain regions. Each dot represents the firing rate of a single unit (non-significant units in grey, significant units in color). Pie charts above display the proportion of significantly modulated units (E: significantly enhanced activity; S: significantly suppressed activity). Solid lines connect the mean values for quiescence (left) and whisking (right) of significantly modulated units. Significant increases in mean population firing rates during whisking are marked by asterisks.

*: $p \leq 0.05$, ***: $p \leq 0.001$; two-tailed paired t-test; n = 413 (BC), 420 (VPM), 261 (POm), and 147 (ZIv) units in total, pooled from n = 6 mice. For exact statistics see Supplementary Table 1.

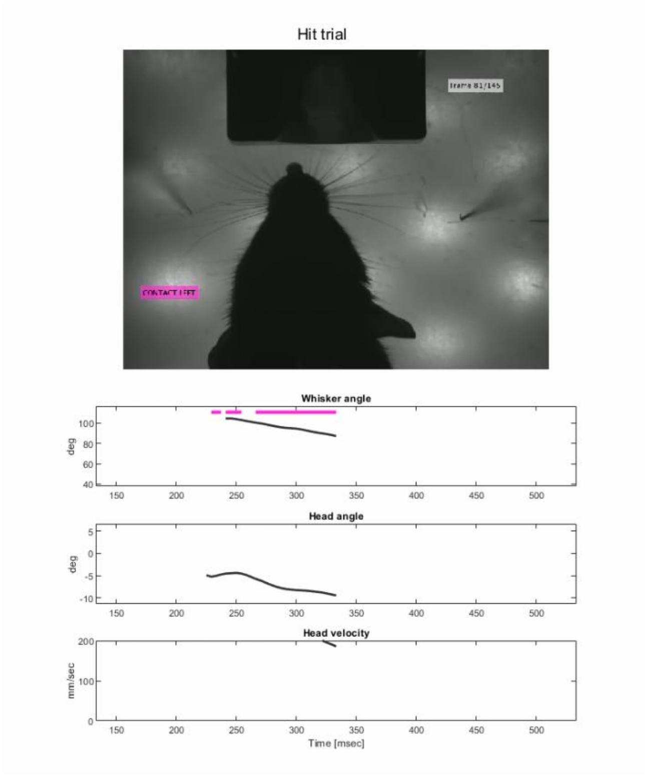

<u>Supplementary video 1:</u> High-speed camera video depicting a hit trial, recorded at 240 FPS and slowed down by a factor of 10 for video playback. The mouse identifies the rewarded aperture and licks lick port to obtain the reward. The accompanying plots display: (1) the average whisker angle relative to the whisker pad (protraction indicated by greater whisker angles), (2) the head angle relative to the midline (values >0° indicate a rightward tilt), and (3) the velocity of the mouse's head within the field of view.

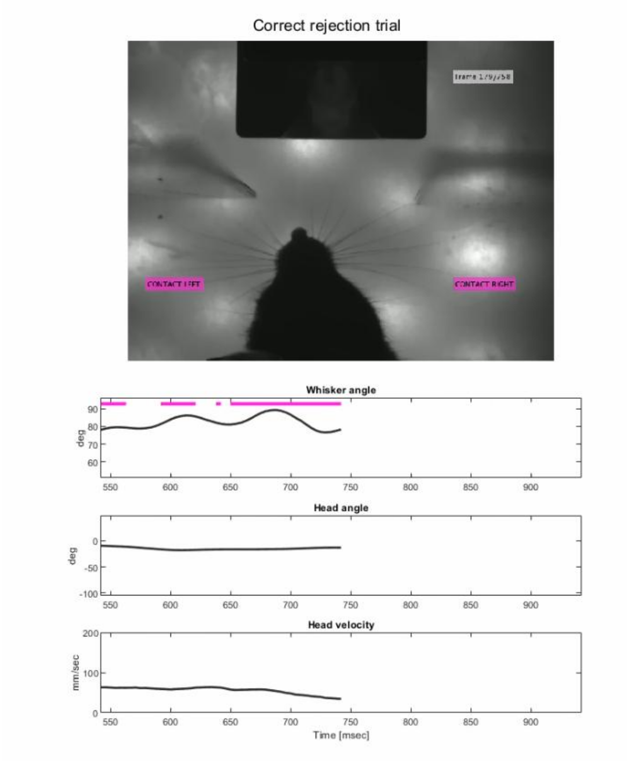

<u>Supplementary video 2:</u> High-speed camera video depicting a correct rejection (CR) trial, recorded at 240 FPS and slowed down by a factor of 10 for video playback. The mouse identifies the punished aperture and turns away from the lick port. The accompanying plots display: (1) the average whisker angle relative to the whisker pad (protraction indicated by greater whisker angles), (2) the head angle relative to the midline (values >0° indicate a rightward tilt), and (3) the velocity of the mouse's head within the field of view.

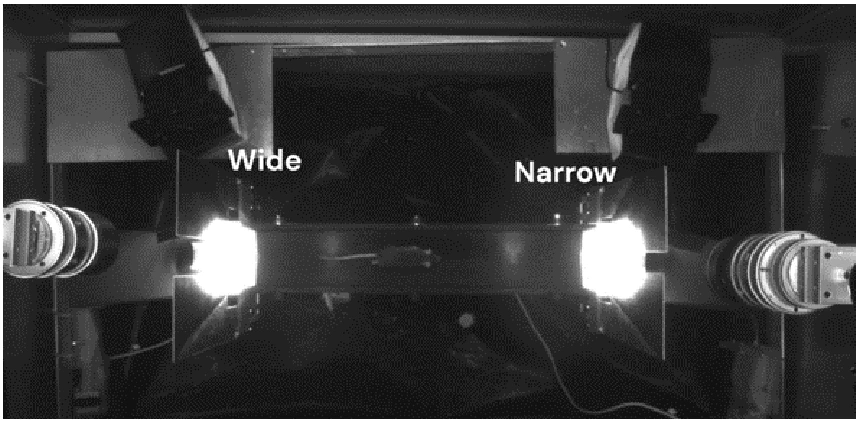

<u>Supplementary video 3:</u> Overview camera video illustrating the experimental setup, recorded at 60 FPS and accelerated by a factor of 2 during video playback. The mouse alternately navigates a linear track to obtain rewards and avoid punishments based on the aperture state. The aperture state, determined by the width of the wing's cleft, changes automatically and randomly between two states. Speakers for administering punishment were positioned behind the linear track. High-speed cameras were positioned overhead at the reward retrieval sites to track whisker-aperture interactions during stimulus sampling.

Figure	Groups	Test statistic, p values, 95% confidence interval (95%CI)	Statistical test
2a	go trials vs. no-go trials	initial rule: $p < 0.05$ for 70%-100% training stage progression<br><br>p values initial rule:<br>bin 21/30 = 0.0242<br>bin 22/30 = 0.0050<br>bin 23/30 = 5.9792×10^{-5}<br>bin 24/30 = 1.8290×10^{-6}<br>bin 25/30 = 1.1331×10^{-7}<br>bin 26/30 = 1.9757×10^{-10}<br>bin 27/30 = 1.5927×10^{-14}<br>bin 28/30 = 1.3803×10^{-14}<br>bin 29/30 = 3.1268×10^{-16}<br>bin 30/30 = 5.8732×10^{-16}<br><br>reversed rule: $p < 0.05$ for 60%-100% training stage progression<br><br>p values reversed rule:<br>bin 3/40 = 0.0430<br>bin 24/40 = 0.0032<br>bin 25/40 = 0.0064<br>bin 26/40 = 0.0103<br>bin 27/40 = 0.0049<br>bin 28/40 = 0.0067<br>bin 29/40 = 0.0110<br>bin 30/40 = 9.2676×10^{-4}	Welch's t-test two-sided

		bin 31/40 = $5.9624x10^{-5}$<br>bin 32/40 = $4.9789x10^{-6}$<br>bin 33/40 = $2.6340x10^{-7}$<br>bin 34/40 = $1.2535x10^{-9}$<br>bin 35/40 = $2.3408x10^{-9}$<br>bin 36/40 = $2.1197x10^{-13}$<br>bin 37/40 = $9.0338x10^{-16}$<br>bin 38/40 = $7.3155x10^{-15}$<br>bin 39/40 = $2.8755x10^{-11}$<br>bin 40/40 = $4.1494x10^{-11}$	
2a	go trials vs. neutral trials	$p < 0.05$ for complete stage<br><br>p values go trials vs. neutral trials:<br>bin 1/8 = 0.012<br>bin 2/8 = $3.3501x10^{-5}$<br>bin 3/8 = $2.9805x10^{-4}$<br>bin 4/8 = $2.0087x10^{-4}$<br>bin 5/8 = $7.2892x10^{-4}$<br>bin 6/8 = $3.2863x10^{-5}$<br>bin 7/8 = $1.5007x10^{-4}$<br>bin 8/8 = $2.0037x10^{-4}$	Welch's t-test two-sided
2a	no-go trials vs. neutral trials	$p < 0.05$ for complete stage<br><br>p values no-go trials vs. neutral trials:<br>bin 1/8 = $4.1667x10^{-13}$<br>bin 2/8 = $5.8623x10^{-15}$<br>bin 3/8 = $3.4958x10^{-10}$<br>bin 4/8 = $3.3040x10^{-10}$<br>bin 5/8 = $2.3633x10^{-6}$<br>bin 6/8 = $3.6030x10^{-8}$<br>bin 7/8 = $1.8910x10^{-10}$<br>bin 8/8 = $7.6511x10^{-9}$	Welch's t-test two-sided
2d	initial vs. reversed rule intercept	t-statistic = -7.1879, p = $1,77x10^{-5}$, 95%CI of the true mean difference = [-825.9572; -438.7095]	two-sided, paired t-test
2d	initial rule vs. second reversal intercept	t-statistic = -5.5581, p = 0.0051, 95%CI of the true mean difference = [-517.9390; -172.8610]	two-sided, paired t-test
2d	initial rule vs. third reversal intercept	t-statistic = -6.2991, p = 0.1002, 95%CI of the true mean difference = [$-1.1118x10^{3}$; 374.8130]	two-sided, paired t-test
2d	reversed rule vs. second reversal intercept	t-statistic = 3.3183, p = 0.0294, 95%CI of the true mean difference = [56.5671; 636.2329]	two-tailed, paired t-test
2d	reversed rule vs. third reversal intercept	t-statistic = 0.6548, p = 0.6309, 95%CI of the true mean difference = [$-4.3988x10^{3}$; $4.8768x10^{3}$]	two-tailed, paired t-test
2d	second vs. third reversal intercept	t-statistic = -0.3057, p = 0.8111, 95%CI of the true mean difference = [$-2.5113x10^{3}$; $2.3933x10^{3}$]	two-tailed, paired t-test

2e	Initial/reversed vs. extinction sessions	Stage (initial rule/reversed rule/extinction): F-statistic = 2171.4110, p = 0.0000, η² = 0.9830<br><br>Initial rule vs. Extinction: mean difference = 2.5820, p = 0.0000, 95%CI = [2.3430, 2.8210]<br>Reversed rule vs. Extinction: mean difference = 2.3010, p = 0.0000, 95%CI = [1.9980, 2.6030]<br><br>Box plots show the median, box limits are the 25th and 75th percentiles, and whiskers extend to the most extreme data points within 1.5× the interquartile range. Data of box plots as follows: minimum; maximum; centre; [lower bound, upper bound] as 25th and 75th percentile; [lower adjacent, upper adjacent].<br>Initial rule: 2.04; 3.52; 2.58; [2.26, 2.93]; [2.04, 3.52]<br>Reversed rule: 1.74; 3.38; 2.21; [1.95, 2.65]; [1.73, 3.38]<br>Extinction: -0.08; 0.13, -0.00, [-0.02, 0.06]; [-0.08, 0.13]	repeated measures anova (two-sided)<br><br>post hoc LSD comparison
2f	Comparison against 0	20 mm: p = 7.2618×10^{-24}<br>16 mm: p = 1.4955×10^{-22}<br>12 mm: p = 7.9206×10^{-26}<br>10 mm: p = 2.1662×10^{-24}<br>8 mm: p = 1.3793×10^{-24}<br>6 mm: p = 8.4266×10^{-22}<br>4 mm: p = 8.3217×10^{-12}<br>2 mm: p = 0.0681<br><br>Box plots show the median, box limits are the 25th and 75th percentiles, and whiskers extend to the most extreme data points within 1.5× the interquartile range. Data of box plots as follows: minimum; maximum; centre; [lower bound, upper bound] as 25th and 75th percentile; [lower adjacent, upper adjacent].<br>20mm: 1.69; 3.46; 2.48; [2.20, 3.09]; [1.69, 3.46]<br>16mm: 1.74; 3.92; 2.68; [2.19, 3.11]; [1.74, 3.92]<br>12mm: 1.96; 3.91; 2.81; [2.53, 3.01]; [1.96, 3.75]<br>10mm: 1.84; 4.06; 2.64; [2.32, 3.04]; [1.84, 4.06]<br>8mm: 1.71; 3.57; 2.45; [2.10, 2.23]; [1.71, 3.42]<br>6mm: 0.48; 2.79; 2.09; [1.76, 2.36]; [0.85, 2.79]<br>4mm: -0.05; 1.49; 0.88; [0.52, 1.15]; [-0.05, 1.49]<br>2mm: -0.57; 0.91; 0.03; [-0.04, 0.25]; [-0.47, 0.68]	one-sample t-test
2g	Initial vs. reversed rule intercept (contrast = 20)	t-statistic = -7.1879, p = $1,77 \times 10^{-5}$, 95%CI of the true mean difference = [-825.9572; -438.7095]<br><br>Box plots show the median, box limits are the 25th and 75th percentiles, and whiskers extend to the most extreme data points within 1.5× the interquartile range. Data of box plots as follows: minimum; maximum; centre; [lower bound, upper bound] as 25th and 75th percentile; [lower adjacent, upper adjacent].<br>Initial rule<br>20mm: 288; 684; 427.50; [383.50, 579]; [288, 684]<br>16mm: 587; 806; 709; [617.50, 781.75]; [587, 806]<br>14mm: 1055; 1427; 1057; [1055.50, 1334.50]; [1055, 1427]<br>12mm: 1133; 1536; 1357; [1288, 1460]; [1133, 1536]<br>Reversed rule<br>20mm: 678; 1581; 1090; [986, 1182]; [692, 1476]<br>16mm: 1396; 2042; 1461; [1412.25, 1896.75]; [1396, 2042]<br>14mm: 2080; 2440; 2260; [2080, 2440]; [2080, 2440]	two-tailed, paired t-test

2g	Initial vs.reversed rule intercept (contrast = 16)	t-statistic = -5.7857, p = 0.0286, 95%CI of the true mean difference = [-1625.68; -238.9859]	two-tailed, paired t-test
3c	Onset latencies upon aperture touch [ms]	Median [IQR]<br>BC: 27.5 [8.5;56.5]<br>VPM: 18.5 [6.5; 43.5]<br>POm: 20.5 [5.5; 46.5]<br>ZIv: 15.5 [5.5; 60.75]<br><br>Mean<br>BC: 40.8308<br>VPM: 35.2179<br>POm: 35.3440<br>ZIv: 37.6185	
4a	Pre pluck vs. post pluck	t-statistic = 13.6362, p = 3.8×10^{-5}, 95%CI of the true mean difference = [1.9665; 2.8802]<br><br>Box plots show the median, box limits are the 25th and 75th percentiles, and whiskers extend to the most extreme data points. Data of box plots as follows: minimum; maximum; centre; [lower bound, upper bound] as 25th and 75th percentile; [lower adjacent, upper adjacent].<br>Pre pluck: 2.17; 3.57; 2.83; [2.32, 3.47]; [2.17, 3.57]<br>Post pluck: -0.06; 1.07; 0.36; [0.16, 0.74]; [-0.06, 1.07]	two-tailed, paired t-test
4b	Pre pluck vs post uni-lateral pluck	6 mm: t-statistic = 12.3257, p = 0.0012, 95%CI of the true mean difference = [1.3847; 2.3486]<br><br>20 mm: t-statistic = 2.9846, p = 0.058, 95%CI of the true mean difference = [-0.0885; 2.7582]<br><br>Box plots show the median, box limits are the 25th and 75th percentiles, and whiskers extend to the most extreme data points. Data of box plots as follows: minimum; maximum; centre; [lower bound, upper bound] as 25th and 75th percentile; [lower adjacent, upper adjacent].<br>6mm<br>Pre: 1.92; 2.47; 2.20; [1.98, 2.41]; [1.92, 2.47]<br>Post: 0.13; 0.53; 0.33; [0.18, 0.48]; [0.13, 0.53]<br>20mm<br>Pre: 2.85; 4.01; 3.28; [3.01, 3.71]; [2.85, 4.01]<br>Post: 1.34; 2.45; 2.15; [1.65, 2.39]; [1.34, 2.45]	two-tailed, paired t-test
4c	Before vs. after sham/ablation	Group (sham/ablation): F-statistic = 4.4580, p = 0.0564, η^2 = 0.1054<br><br>Condition (before/after): F-statistic = 19.6032, p = 8.2×10^{-4}, η^2 = 0.4637<br><br>Sham before vs. Ablation after: mean difference = 1.7980, p = 0.0028, 95%CI = [0.6436, 2.9525]<br>Sham after vs. Ablation after: mean difference = 1.9030, p = 0.0018, 95%CI = [0.7485, 3.0575]<br>Ablation before vs. ablation after: mean difference = 1.2662, p = 0.0303, 95%CI = [0.1117, 2.4207]<br><br>Box plots show the median, box limits are the 25th and 75th percentiles, and whiskers extend to the most extreme data points. Data of box plots as follows: minimum; maximum; centre; [lower bound, upper bound] as 25th and 75th percentile; [lower adjacent, upper adjacent].<br>Before BC ablation<br>Sham: 2.18; 3.59; 3.17; [2.54, 3.51]; [2.18, 3.59]	two-way anova<br><br>Tukey's HSD test for multiple comparisons

		Ablation: 2.83; 3.43; 3.13; [2.93, 3.34]; [2.83, 3.43]<br>After BC ablation<br>Sham: 1.66; 3.35; 2.49; [1.81, 3.18]; [1.66, 3.35]<br>Ablation: 0.91; 1.58; 1.22; [1.01, 1.45]; [0.91, 1.58]	
4e	Maximum d prime sham vs. ablation	t-statistic = 3.1176, p = 0.0206, 95%CI of the true mean difference = [0.1933; 1.6038]<br><br>Box plots show the median, box limits are the 25th and 75th percentiles, and whiskers extend to the most extreme data points. Data of box plots as follows: minimum; maximum; centre; [lower bound, upper bound] as 25th and 75th percentile; [lower adjacent, upper adjacent].<br>Sham: 3.16; 4.01; 3.36; [3.20, 3.74]; [3.16, 4.01]<br>BC ablated: 2.10; 3.10; 2.55; [2.24, 2.90]; [2.10, 3.10]	two-tailed, independent samples t-test
4g	Bepanthen vs. lidocaine sessions	t-statistic = 3.9892, p = 0.0282, 95%CI of the true mean difference = [0.3585; 3.1870]<br><br>Box plots show the median, box limits are the 25th and 75th percentiles, and whiskers extend to the most extreme data points. Data of box plots as follows: minimum; maximum; centre; [lower bound, upper bound] as 25th and 75th percentile; [lower adjacent, upper adjacent].<br>Control: 1.46; 3.45; 2.85; [2.26, 3.20]; [1.46, 3.45]<br>Lidocaine: -0.35; 1.97; 1.21; [0.25, 1.53]; [-0.35, 1.97]	two-tailed paired t-test with means for each animal
4i	Lidocaine sessions<br>Control sessions<br>Shuffled labels	Mean AUC = 0.8687, 95%CI = [0.8207, 0.9050]<br>Mean AUC = 0.9059, 95%CI = [0.8590, 0.9304]<br>Mean AUC = 0.5303, 95%CI = [0.4580, 0.6216]	
4i	Lidocaine vs. control	t-statistic = -1.6676, p = 0.0972	two-tailed, two-sample t-test
4i	Control vs. shuffled	t-statistic = 7.7886, p = 3.5392×10^{-12}	two-tailed, two-sample t-test
4i	Lidocaine vs. shuffled	t-statistic = 6.7526, p = 4.4247×10^{-10}	two-tailed, two-sample t-test
4j	BC	t-statistic = 9.2115, p = 9.2337×10^{-5}, 95%CI of the true mean difference = [15.9986; 27.5728]<br><br>Box plots show the median, box limits are the 25th and 75th percentiles, and whiskers extend to the most extreme data points. Data of box plots as follows: minimum; maximum; centre; [lower bound, upper bound] as 25th and 75th percentile; [lower adjacent, upper adjacent].<br>Control: 74; 88.5; 79; [74.75, 81.5]; [74, 88.5]<br>Lidocaine: 55.5; 61; 57; [56.12, 58.75]; [55.5, 61]	two-tailed, paired t-test
4j	VPM	t-statistic = 8.4932, p = 1.4578×10^{-4}, 95%CI of the true mean difference = [10.2717; 18.5855]	two-tailed, paired t-test

		Box plots show the median, box limits are the 25th and 75th percentiles, and whiskers extend to the most extreme data points. Data of box plots as follows: minimum; maximum; centre; [lower bound, upper bound] as 25th and 75th percentile; [lower adjacent, upper adjacent].<br>Control: 67; 78.5; 73.5; [68.25,76.88]; [67, 78.5]<br>Lidocaine: 54.5; 63; 56.5; [55.75, 62.38]; [54.5, 63]	
4j	POm	t-statistic = 3.2869, p = 0.0167, 95%CI of the true mean difference = [2.7197; 18.5660]<br><br>Box plots show the median, box limits are the 25th and 75th percentiles, and whiskers extend to the most extreme data points. Data points beyond the whiskers are plotted individually as outliers. Data of box plots as follows: minimum; maximum; centre; [lower bound, upper bound] as 25th and 75th percentile; [lower adjacent, upper adjacent].<br>Control: 62; 76.5; 72; [65, 74.75]; [62, 76.5]<br>Lidocaine: 51.5; 63; 59.5; [58, 61.75]; [57.5, 63]	two-tailed, paired t-test
4j	ZIv	t-statistic = 4.1617, p = 0.0059, 95%CI of the true mean difference = [4.4735; 17.2408]<br><br>Box plots show the median, box limits are the 25th and 75th percentiles, and whiskers extend to the most extreme data points. Data points beyond the whiskers are plotted individually as outliers. Data of box plots as follows: minimum; maximum; centre; [lower bound, upper bound] as 25th and 75th percentile; [lower adjacent, upper adjacent].<br>Control: 61; 78; 72.5; [66.37, 74.38]; [61, 78]<br>Lidocaine: 55.5; 64.5; 60; [59.12, 61]; [59, 61]	two-tailed, paired t-test
5b	Comparison of retraction times [s]	Median [Q1; Q3] =<br>6.91 [3.64; 27.03] (chance)<br>1.78 [0.83; 2.95] (good)<br>1.27 [0.56; 2.43] (expert)<br><br>p =<br>0.0268 (expert vs. good)<br>2.5879×10^{-6} (expert vs. chance)<br><br>Box plots show the median, box limits are the 25th and 75th percentiles, and whiskers extend to the most extreme data points. Data of box plots as follows: minimum; maximum; centre; [lower bound, upper bound] as 25th and 75th percentile; [lower adjacent, upper adjacent].<br>Chance: 0.296; 480.478; 6.9135; [3.635, 27.0325]; [0.296, 30.215]<br>Good: 0.491; 46.615; 1.779; [0.8253, 2.9532]; [0.491, 5.048]<br>Expert: 0.146; 75.551; 1.274; [0.5628, 2.492]; [0.146, 5.228]	two-tailed Wilcoxon rank sum test
5c	Comparison of retraction distances [mm]	Mean ± 95%CI =<br>2.75±7.51 (chance)<br>-0.31±1.49 (good)<br>-2.00±0.41 (expert)<br><br>p =<br>0.2251 (expert vs. good)<br>0.0277 (expert vs. chance)	two-tailed Wilcoxon rank sum test

5e	Lick latencies in the different trial quarters of the initial learning stage - Go trials	Chi-square = 327.82, p = 9.4617×10^{-71}<br><br>Quarter 1 vs. quarter 2, p = 1.3660×10^{-8}<br>Quarter 1 vs. quarter 3, p = 0.0000<br>Quarter 1 vs. quarter 4, p = 0.0000<br>Quarter 2 vs. quarter 3, p = 1.6459×10^{-7}<br>Quarter 2 vs. quarter 4, p = 0.0000<br>Quarter 3 vs. quarter 4, p = 7.3177×10^{-8}<br><br>Box plots show the median, box limits are the 25th and 75th percentiles, and whiskers extend to the most extreme data points. Data points beyond the whiskers are plotted individually as outliers. Data of box plots as follows: minimum; maximum; centre; [lower bound, upper bound] as 25th and 75th percentile; [lower adjacent, upper adjacent].	two-tailed Friedman test<br><br>Dunn-Sidak-adjusted multiple comparisons
5e	Lick latencies in the different trial quarters of the initial learning stage - No-Go trials	Chi-square = 85.6, p = 1.9246×10^{-18}<br><br>Quarter 1 vs. quarter 2, p = 5.4231×10^{-11}<br>Quarter 1 vs. quarter 3, p = 1.3276×10^{-5}<br>Quarter 1 vs. quarter 4, p = 0.8513<br>Quarter 2 vs. quarter 3, p = 0.2014<br>Quarter 2 vs. quarter 4, p = 1.4655×10^{-14}<br>Quarter 3 vs. quarter 4, p = 3.3031×10^{-8}<br><br>Box plots show the median, box limits are the 25th and 75th percentiles, and whiskers extend to the most extreme data points. Data points beyond the whiskers are plotted individually as outliers. Data of box plots as follows: minimum; maximum; centre; [lower bound, upper bound] as 25th and 75th percentile; [lower adjacent, upper adjacent].<br>Go trials<br>Q1: 201; 893466; 492; [416, 621]; [201, 928.50]<br>Q2: 262; 2489; 435; [383, 526.50]; 262, 741.75]<br>Q3: 241; 2274; 388.50; [339, 474]; [241, 676.50]<br>Q4: 197; 1861; 343; [303, 405]; [197, 558]<br>No-Go trials<br>Q1: 327; 726438; 630; [491.75, 855.50]; [327, 1401.13]<br>Q2: 327; 71154; 535.50; [451, 638]; [327, 918.50]<br>Q3: 319; 13509; 568; [474, 723.25]; [319, 1097.13]<br>Q4: 310; 16536; 649; [541.75, 922.75]; [310, 1494.25]	two-tailed Friedman test<br><br>Dunn-Sidak-adjusted multiple comparisons
5g	Naive CR vs. naive FA	p = 2.3356×10^{-8}	two-tailed Wilcoxon rank sum test
5g	Expert CR vs. expert FA	p = 0.5762	two-tailed Wilcoxon rank sum test
5g	Naive CR vs. expert CR	p = 1.7981×10^{-6}	two-tailed Wilcoxon rank sum test
5g	Naive FA vs. expert FA	p = 0.2900	two-tailed Wilcoxon rank sum test
5k	Firing rates during rest and locomotion	Significantly enhanced cells: 21/413 (BC), 66/420 (VPM), 42/261 (POm), 17/147 (ZIv)<br>Significantly suppressed cells: 30/413 (BC), 24/420 (VPM), 10/261 (POm), 4/147 (ZIv)	two-tailed paired t-test

		Mean rest firing rate [Hz]: 17.21 (BC), 18.71 (VPM), 12.81 (POm), 6.64 (ZIv)<br>Mean locomotion firing rate [Hz]: 14.93 (BC), 30.67 (VPM), 26.98 (POm), 20.57 (ZIv)<br><br>p = 0.33 (BC), 4.1478×10^{-5} (VPM), 4.4448×10^{-7} (POm), 0.0036 (ZIv)	
6f	Proportions of tuned units (egocentric)	11.2±8.7% in BC,<br>6.8±4.2% in VPM,<br>5.4±5.5% in POm,<br>7.2±5.6% in ZIv	
6f	Proportions of tuned units (allocentric)	12.0±8.8% in BC,<br>6.2±4.6% in VPM,<br>6.2±5.2% in POm,<br>1.7±2.4% in ZIv	
S1c	Naive vs. expert trials per sessions	initial rule: t-statistic = -5.4562, p = 1.99×10^{-4}, 95%CI of the true mean difference = [-61.369; -26.0893]<br><br>reversed rule: t-statistic = -1.6198, p = 0.1336, 95%CI of the true mean difference = [-10.8114; 1.6447]	two-tailed, paired t-test
S1e	Initial vs. reversed rule learning speed	t-statistic = 3.4331, p = 0.0056, 95%CI of the true mean difference = [0.0012; 0.0053]	two-tailed, paired t-test
S4a	Performance upon OB lesion	Mean ± Std<br><br>Normal textures: 2.61±0.28<br><br>Inverted textures: 1.80±1.03<br><br>Inverted textures (post-injection)<br>Sham: 2.68±0.68<br>NMDA: 0.17±0.36<br>p = 3.6534×10^{-4}<br><br>Normal textures (post-injection)<br>Sham: 3.29±0.50<br>NMDA: 1.87±0.58<br>p = 5.0056×10^{-4}<br><br>p NMDA inverted vs. normal = 2.4831e-04<br><br>p Sham inverted vs. normal = 0.0886<br><br>*p-values are Bonferroni corrected*	two-tailed Wilcoxon rank sum test
S4b	Backlights on vs. backlights off	F-statistic = 0.009, p = 0.926, 95%CI of the true mean difference = [-0.728; 0.488]	repeated measures anova
S4c	Two vs. one wing remaining	t-statistic = 8.9381, p = 3.1088×10^{-8}, 95%CI of the true mean difference = [1.3640; 2.1982]	two-tailed, paired t-test
S4e	Proportion of trials with performance drop	Mean ± 95%CI<br>Control: 0.28 ± 0.01<br>Ablated: 0.21 ± 0.03<br><br>p = 0.0286	two-tailed Wilcoxon rank sum test

S4f	Residuals of the sigmoidal fit in ablated and native mice	Median [IQR]<br>Control: 0.15 [0.07;0.27]<br>Ablated: 0.26 [0.10; 0.44]<br><br>Mean ± 95%CI<br>Control: 0.1909 ± 0.0058<br>Ablated: 0.2849 ± 0.0036<br><br>$p = 3.2132x10^{-96}$	two-tailed Wilcoxon rank sum test
S4g	Averaged whisker angles during wide and narrow trials	Significant time window, with Bonferroni correction, starting at 70 ms after trigger onset.	Welch's t-test
S5	Firing rates during quiescence and whisking	Significantly enhanced cells: 60 (BC), 101 (VPM), 81 (POm), 36 (ZIv)<br>Significantly suppressed cells: 20 (BC), 6 (VPM), 2 (POm), 5 (ZIv)<br><br>Mean quiescence firing rate [Hz]: 13.43 (BC), 16.13 (VPM), 18.53 (POm), 13.74 (ZIv)<br>Mean whisking firing rate [Hz]: 16.96 (BC), 23.76 (VPM), 27.45 (POm), 19.08 (ZIv)<br><br>$p = 2.7496x10^{-16}$ (BC), $1.1239x10^{-46}$ (VPM), $2.1961x10^{-35}$ (POm), $7.5269x10^{-16}$ (ZIv)	two-tailed paired t-test

Supplementary table 1. Description of statistical parameters.
