# Supplementary material for: A tactile discrimination task to study neuronal dynamics in freely-moving mice
Source: Nat Commun. 2025 Jul 11;16:6421. doi: 10.1038/s41467-025-61792-0 (PMC12254278; doi:10.1038/s41467-025-61792-0)
Supplement: Supplementary file 2 — Description of Additional Supplementary Files [file 41467_2025_61792_MOESM2_ESM.pdf]

Description of Additional Supplementary Files

Title: Supplementary Video 1

Description: High-speed camera video depicting a hit trial, recorded at 240 FPS and slowed down by a factor of 10 for video playback. The mouse identifies the rewarded aperture and licks lick port to obtain the reward. The accompanying plots display: (1) the average whisker angle relative to the whisker pad (protraction indicated by greater whisker angles), (2) the head angle relative to the midline (values >0° indicate a rightward tilt), and (3) the velocity of the mouse's head within the field of view.

Title: Supplementary Video 2

Description: High-speed camera video depicting a correct rejection (CR) trial, recorded at 240 FPS and slowed down by a factor of 10 for video playback. The mouse identifies the punished aperture and turns away from the lick port. The accompanying plots display: (1) the average whisker angle relative to the whisker pad (protraction indicated by greater whisker angles), (2) the head angle relative to the midline (values >0° indicate a rightward tilt), and (3) the velocity of the mouse's head within the field of view.

Title: Supplementary Video 3

Description: Overview camera video illustrating the experimental setup, recorded at 60 FPS and accelerated by a factor of 2 during video playback. The mouse alternately navigates a linear track to obtain rewards and avoid punishments based on the aperture state. The aperture state, determined by the width of the wing’s cleft, changes automatically and randomly between two states. Speakers for administering punishment were positioned behind the linear track. High-speed cameras were positioned overhead at the reward retrieval sites to track whisker-aperture interactions during stimulus sampling.
