# Supplementary material for: A tactile discrimination task to study neuronal dynamics in freely-moving mice
Source: Nat Commun. 2025 Jul 11;16:6421. doi: 10.1038/s41467-025-61792-0 (PMC12254278; doi:10.1038/s41467-025-61792-0)
Supplement: Supplementary file 6 — Reporting Summary [file 41467_2025_61792_MOESM6_ESM.pdf]

nature portfolio

Corresponding author(s): Alexander Groh

Last updated by author(s): Jun 5, 2025

Reporting Summary

Nature Portfolio wishes to improve the reproducibility of the work that we publish. This form provides structure for consistency and transparency in reporting. For further information on Nature Portfolio policies, see our Editorial Policies and the Editorial Policy Checklist.

Statistics

For all statistical analyses, confirm that the following items are present in the figure legend, table legend, main text, or Methods section.

n/a	Confirmed	
☐	☒	The exact sample size (*n*) for each experimental group/condition, given as a discrete number and unit of measurement
☐	☒	A statement on whether measurements were taken from distinct samples or whether the same sample was measured repeatedly
☐	☒	The statistical test(s) used AND whether they are one- or two-sided<br>*Only common tests should be described solely by name; describe more complex techniques in the Methods section.*
☒	☐	A description of all covariates tested
☐	☒	A description of any assumptions or corrections, such as tests of normality and adjustment for multiple comparisons
☐	☒	A full description of the statistical parameters including central tendency (e.g. means) or other basic estimates (e.g. regression coefficient) AND variation (e.g. standard deviation) or associated estimates of uncertainty (e.g. confidence intervals)
☐	☒	For null hypothesis testing, the test statistic (e.g. *F*, *t*, *r*) with confidence intervals, effect sizes, degrees of freedom and *P* value noted<br>*Give P values as exact values whenever suitable.*
☒	☐	For Bayesian analysis, information on the choice of priors and Markov chain Monte Carlo settings
☒	☐	For hierarchical and complex designs, identification of the appropriate level for tests and full reporting of outcomes
☐	☒	Estimates of effect sizes (e.g. Cohen's *d*, Pearson's *r*), indicating how they were calculated

Our web collection on statistics for biologists contains articles on many of the points above.

Software and code

Policy information about availability of computer code

Data collection

Data acquisition during behavior was done with Syntalos (Klumpp et al., 2024). An intan-module recorded all analog signals from the lick ports. The overview camera was connected to a recorder module that operated continuously throughout the session. The high-speed cameras and an event list were controlled by a custom-made Python script. The cameras were programmed to start recording when the animal approached the corresponding LP, determined by the crossing of the corresponding light beam. The event list kept track of the triggered light beams and reported the state of the apertures. The list also noted if a trial was a success or failure.

Data analysis

The behavioral data was analyzed using custom code developed in MATLAB R2023a (The MathWorks, Inc.), which is available on A-tactile-discrimination-task-to-study-neuronal-dynamics-in-freely-moving-mice/DataAndScripts at main · GrohLab/A-tactile-discrimination-task-to-study-neuronal-dynamics-in-freely-moving-mice · GitHub (Heimburg, F., Saluti, N. M., Timm, J., Adlakha, A., Castelanelli, M., Klumpp M., Embray L., Both M., Draguhn A., Kuner T., Groh A., n.d.). P-values ≤ 0.05 were considered significant. Mean values were reported with standard deviations and median values with interquartile range (IQR).

Statistical analysis of the learning process
Task performance in each session was measured using the discriminability index d-prime (d'), also known as Fisher discrimination index (Macmillan & Douglas Creelman, 1991; Stanislaw & Todorov, 1999). The d' is a statistical measure to quantify the ability to discriminate between signal and noise and is defined as the difference between the z-scores of the hit rate and the FA rate: d' = z(hit rate)−z(false alarm rate). Due to the constraints of the z-score transformation (which cannot handle proportions of 0 or 1), hit rates and FA rates of 0 are typically adjusted to (12 * n) and rates of 1 are adjusted to (1-12 * n), with n being the number of go- (for hit rate adjustment), or no-go trials (for FA rate adjustment), respectively. A higher d' indicates greater discriminability, meaning the observer can more effectively distinguish signal from noise. A d' of 0 describes a discrimination performance at chance level. An animal was considered to reach expert performance when it

crossed a d′ of 1.65, corresponding to a one-tailed significance level of α = 0.05.

Extraction of behavioral data from videos
Spatial information of the mice was extracted from video recordings using DeepLabCut (Mathis et al., 2018). Following preprocessing with median background image subtraction, the DeepLabCut model was trained on a subset of manually annotated video frames. For the high-speed videos, these markers consisted of 8 data points for two whiskers on each side from whisker base to tip and markers for the mouse's head contours. In overview videos, spatial positioning and velocity were estimated based on anatomical landmarks on the mouse's body and head. For the positional data analysis (Fig. 4D), the linear track was divided into rectangles measuring 10 mm by 1 mm. The time spent in each area was calculated by counting the number of video frames in which the mouse was present within each specific rectangle.

Electrophysiological data
The raw electrophysiological data was spike-sorted with KiloSort 2.0. Subsequently, the sorted clusters were curated automatically using the Ecephys spike sorting algorithm provided by the Allen Brain Observatory (code available at https://github.com/alleninstitute/ecephys_spike_sorting). The algorithm includes noise templates to identify and exclude clusters exhibiting characteristics indicative of noise, such as irregular waveform shapes and interspike interval (ISI) histograms. The following quality metrics were then applied to further exclude any clusters indicative of multi-unit activity: isolation distance (computed from Mahalanobis distance) greater than 15 and ISI violation below 3 %, with an ISI threshold of 1.5 milliseconds. The criteria were selected based on previous studies (Onodera & Kato, 2022; Rosenberg & Horn, 2016; Trautmann et al., 2019), where they have been demonstrated to effectively distinguish well-isolated single units from noise and multi-unit activity. The individual units were further classified into fast spiking (FS) and regular spiking (RS) units, putatively representing inhibitory and excitatory neurons, respectively (Barthó et al., 2004). The threshold for differentiating RS from FS units was set to a trough-to-peak duration of 350 µs for cortex and zona incerta, and 300 µs for thalamic nuclei. These thresholds align with previous studies (Barthó et al., 2014; Mahrach et al., 2020) and consider the fact that extracellular waveforms in the thalamus are shorter than those in the cortex (Barthó et al., 2014). For further analysis, only RS units were selected for BC, VPM, and POm, while FS units were selected for ZIv.

Extraction of Calcium Traces and Spatial Footprints
The preprocessing, motion correction, and extraction of the calcium traces and spatial footprints from the videos was done with Minian (Dong et al., 2022). All downstream processing was done using custom code developed in MATLAB R2023a (The MathWorks, Inc.).

Touch modulated neurons
Calcium transients of the POm neurons were extracted in a window of 400 ms before and after the whisker touch (see above). The summed calcium activity before and after the touch was compared with a paired two-tailed t-test. Whisker touch responsiveness for the extracellular recordings was determined by a statistically significant increase in firing rates (to either of the two aperture states) within a 200 ms response window following aperture touch, compared to a baseline window preceding touch onset. Statistical significance was determined by a two-sample Kolmogorov-Smirnov test. Those units which showed significant differences were identified as touch-modulated neurons.

Decoding of aperture width form unit spike trains
Aperture width decoding from unit spike trains traces was conducted using the Neural Decoding Toolbox (Meyers, 2013). The dataset was partitioned into a training set (90% of labels) and a test set (10% of labels). Each set contained the spike traces of individual units for a given trial and the corresponding aperture labels. A support vector machine (SVM) classifier was trained using the LIBSVM software package. The classifier was subjected to a 10-fold cross-validation, where it was trained and tested on different data partitions. The mean decoding accuracy was then calculated over a window from trigger onset to 400 ms post-trigger onset.

Decoding of aperture width from whisker angles
Aperture width decoding from whisker angles was performed using MATLAB's built-in generalized additive model (GAM) classifier. The dataset was partitioned into a training set (80% of labels) and a test set (20% of labels). Each set included whisker angles recorded 20 frames before and 100 frames after whisker touch, with a sampling rate of 240 fps, along with the corresponding aperture labels. In the shuffled decoding accuracy analysis, the whisker angle traces were randomly allocated to an aperture label. P-values were determined using pointwise 95% confidence intervals, which were computed by the MATLAB "rocmetrics" function based on 100 pointwise bootstrap resamples.

For manuscripts utilizing custom algorithms or software that are central to the research but not yet described in published literature, software must be made available to editors and reviewers. We strongly encourage code deposition in a community repository (e.g. GitHub). See the Nature Portfolio guidelines for submitting code & software for further information.

Data

Policy information about availability of data

All manuscripts must include a data availability statement. This statement should provide the following information, where applicable:

- Accession codes, unique identifiers, or web links for publicly available datasets
- A description of any restrictions on data availability
- For clinical datasets or third party data, please ensure that the statement adheres to our policy

All the data are in the manuscript or in supplementary material. Source data and code ist available under the following link: https://github.com/GrohLab/A-tactile-discrimination-task-to-study-neuronal-dynamics-in-freely-moving-mice/tree/v1.0

Research involving human participants, their data, or biological material

Policy information about studies with human participants or human data. See also policy information about sex, gender (identity/presentation), and sexual orientation and race, ethnicity and racism.

Reporting on sex and gender

Use the terms sex (biological attribute) and gender (shaped by social and cultural circumstances) carefully in order to avoid confusing both terms. Indicate if findings apply to only one sex or gender; describe whether sex and gender were considered in study design; whether sex and/or gender was determined based on self-reporting or assigned and methods used. Provide in the source data disaggregated sex and gender data, where this information has been collected, and if consent has

	been obtained for sharing of individual-level data; provide overall numbers in this Reporting Summary. Please state if this information has not been collected.<br>*Report sex- and gender-based analyses where performed, justify reasons for lack of sex- and gender-based analysis.*
Reporting on race, ethnicity, or other socially relevant groupings	*Please specify the socially constructed or socially relevant categorization variable(s) used in your manuscript and explain why they were used. Please note that such variables should not be used as proxies for other socially constructed/relevant variables (for example, race or ethnicity should not be used as a proxy for socioeconomic status).*<br>*Provide clear definitions of the relevant terms used, how they were provided (by the participants/respondents, the researchers, or third parties), and the method(s) used to classify people into the different categories (e.g. self-report, census or administrative data, social media data, etc.)*<br>*Please provide details about how you controlled for confounding variables in your analyses.*
Population characteristics	*Describe the covariate-relevant population characteristics of the human research participants (e.g. age, genotypic information, past and current diagnosis and treatment categories). If you filled out the behavioural & social sciences study design questions and have nothing to add here, write "See above."*
Recruitment	*Describe how participants were recruited. Outline any potential self-selection bias or other biases that may be present and how these are likely to impact results.*
Ethics oversight	*Identify the organization(s) that approved the study protocol.*

Note that full information on the approval of the study protocol must also be provided in the manuscript.

Field-specific reporting

Please select the one below that is the best fit for your research. If you are not sure, read the appropriate sections before making your selection.

☒ Life sciences ☐ Behavioural & social sciences ☐ Ecological, evolutionary & environmental sciences

For a reference copy of the document with all sections, see nature.com/documents/nr-reporting-summary-flat.pdf

Life sciences study design

All studies must disclose on these points even when the disclosure is negative.

Sample size	Sample sizes were chosen in accordance with standard numbers of animals in comparable studies in the field, e.g. Aoki, R., Tsubota, T., Goya, Y. et al. An automated platform for high-throughput mouse behavior and physiology with voluntary head-fixation. Nat Commun 8, 1196 (2017). https://doi.org/10.1038/s41467-017-01371-0; Danilo La Terra, Ann-Sofie Bjerre, Marius Rosier, Rei Masuda, Tomás J Ryan, Lucy M Palmer (2022) The role of higher-order thalamus during learning and correct performance in goal-directed behavior eLife 11:e77177, https://doi.org/10.7554/eLife.77177<br><br>The exact size of specific experimental groups are given in the figures legends, results and methods.
Data exclusions	Tetrode tracks were manually reconstructed from the microscope images using the Amira software (v6.5, Thermo Fisher Scientific, Waltham, MA). Subsequently, all tetrodes located outside of the designated target areas were excluded from further analysis.<br><br>The raw electrophysiological data was spike-sorted with KiloSort 2.0. Subsequently, the sorted clusters were curated automatically using the Ecephys spike sorting algorithm provided by the Allen Brain Observatory (code available at https://github.com/alleninstitute/ecephys_spike_sorting). The algorithm includes noise templates to identify and exclude clusters exhibiting characteristics indicative of noise, such as irregular waveform shapes and interspike interval (ISI) histograms. The following quality metrics were then applied to further exclude any clusters indicative of multi-unit activity: isolation distance (computed from Mahalanobis distance) greater than 15 and ISI violation below 3 %, with an ISI threshold of 1.5 milliseconds. The criteria were selected based on previous studies (Onodera & Kato, 2022; Rosenberg & Horn, 2016; Trautmann et al., 2019), where they have been demonstrated to effectively distinguish well-isolated single units from noise and multi-unit activity. The individual units were further classified into fast spiking (FS) and regular spiking (RS) units, putatively representing inhibitory and excitatory neurons, respectively (Barthó et al., 2004). The threshold for differentiating RS from FS units was set to a trough-to-peak duration of 350 µs for cortex and zona incerta, and 300 µs for thalamic nuclei. These thresholds align with previous studies (Barthó et al., 2014; Mahrach et al., 2020) and consider the fact that extracellular waveforms in the thalamus are shorter than those in the cortex (Barthó et al., 2014). For further analysis, only RS units were selected for BC, VPM, and POm, while FS units were selected for ZIv.
Replication	All experiments were replicated in at least 5 mice with a few exceptions: Figure 2G, 4 mice were used for this experiment. Figure 3B, 3 mice were trained on each the 16 - and 14 mm contrast.
Randomization	Each experiment used a cohort of litter-mates in which all mice were exposed to the same conditions and underwent the same behavioral stages. The mouse ID # was assigned randomly to each mouse in a new cohort. In case it was necessary to divide mice from the same cohort into two different groups with different conditions, the mice were randomly assigned to groups based on their mouse IDs, which were randomly assigned at the start.
Blinding	Recordings and analysis were run automatically by a computer with predefined parameters, which removes any subjective components of the data collection.

Reporting for specific materials, systems and methods

We require information from authors about some types of materials, experimental systems and methods used in many studies. Here, indicate whether each material, system or method listed is relevant to your study. If you are not sure if a list item applies to your research, read the appropriate section before selecting a response.

Materials & experimental systems

n/a	Involved in the study
☒	☐ Antibodies
☒	☐ Eukaryotic cell lines
☒	☐ Palaeontology and archaeology
☐	☒ Animals and other organisms
☒	☐ Clinical data
☒	☐ Dual use research of concern
☒	☐ Plants

Methods

n/a	Involved in the study
☒	☐ ChIP-seq
☒	☐ Flow cytometry
☒	☐ MRI-based neuroimaging

Animals and other research organisms

Policy information about studies involving animals; ARRIVE guidelines recommended for reporting animal research, and Sex and Gender in Research

Laboratory animals	The experiments were done with adult male mice from the inbred strain C57BL/6NRj (Janvier Labs, Le Genest-Saint-Isle, France). Mice were 8-10 weeks old at the start of training, with a mean body weight of 25.94 ± 2.33 g (mean ± standard deviation) at the start of the experiments. Mice were separately housed in a ventilated Scantainer (Scantainer Classic, SCANBUR A/S, Karlslunde, Denmark) under a 12-hour inverted light/dark cycle (lights off at 7:00 a.m. and on at 7:00 p.m.) at a controlled temperature (22-25 °C) and humidity (40-60 %). During behavioral training, mice were held on a food restriction schedule, and their body weight was maintained at 95-85% of their initial body weight. Under the food restriction schedule, mice were given dry food pellets (10-20 % of their body weight) daily at the end of behavioral training. Water was available ad libitum.
Wild animals	No wild animals were used in this study.
Reporting on sex	The experiments were done with adult male mice. Sex-specific effects were not addressed in this study.
Field-collected samples	No field-collected samples were used in this study.
Ethics oversight	All experimental procedures were approved by the local governing body (Regierungspräsidium Karlsruhe, Germany, approval numbers: 35-9185.81/G-216/19 and 35-9185.82/A-8/20) and performed according to their ethical guidelines.

Note that full information on the approval of the study protocol must also be provided in the manuscript.

Plants

Seed stocks	*Report on the source of all seed stocks or other plant material used. If applicable, state the seed stock centre and catalogue number. If plant specimens were collected from the field, describe the collection location, date and sampling procedures.*
Novel plant genotypes	*Describe the methods by which all novel plant genotypes were produced. This includes those generated by transgenic approaches, gene editing, chemical/radiation-based mutagenesis and hybridization. For transgenic lines, describe the transformation method, the number of independent lines analyzed and the generation upon which experiments were performed. For gene-edited lines, describe the editor used, the endogenous sequence targeted for editing, the targeting guide RNA sequence (if applicable) and how the editor was applied.*
Authentication	*Describe any authentication procedures for each seed stock used or novel genotype generated. Describe any experiments used to assess the effect of a mutation and, where applicable, how potential secondary effects (e.g. second site T-DNA insertions, mosiacism, off-target gene editing) were examined.*
